# Correction: Prevalence, clinical management and risks associated with acute faecal incontinence in the critical care setting: The FIRST questionnaire survey

**DOI:** 10.1186/cc10225

**Published:** 2011-05-19

**Authors:** C Bayón García, R Binks, E De Luca, C Dierkes, A Franci, E Gallart, G Niederalt, D Wyncoll

**Affiliations:** 1 Hospital Universitario Ramón y Cajal, Madrid, Spain; 2 Airedale NHS Foundation Trust, West Yorkshire, UK; 3 Policlinico Tor Vergata, Rome, Italy; 4 University of Regensburg, Regensburg, Germany; 5 San Giovanni di Dio Hospital, Florence, Italy; 6 Hospital Universitari Vall d'Hebron, Barcelona, Spain; 7 Guy's & St Thomas' NHS Foundation Trust, London, UK

## Correction

After the publication of this abstract [1], we found that not all authors were listed. A full author list is provided here. We regret any inconvenience this error may have caused.
